# Supplementary material for: Adults with depressive symptoms have lower odds of dietary supplement use
Source: PLoS One. 2024 May 8;19(5):e0302637. doi: 10.1371/journal.pone.0302637 (PMC11078386; doi:10.1371/journal.pone.0302637)
Supplement: S1 Table — (DOCX) [file pone.0302637.s001.docx]

**Table 1S.**

| Outcome | Depressive symptoms | | Depressive symptoms severity | |
| --- | --- | --- | --- | --- |
|  | aCoef. Estm. (95% CI) | *P* value | aCoef. Estm. (95% CI) | *P* value |
| Vitamin B1 | -1.637 (-10.997, 7.723) | 0.729 | 0.440 ( 0.126, 0.754) | 0.007 |
| Vitamin B2 | -0.703 (-4.080, 2.675) | 0.680 | 0.113 (-0.003, 0.229) | 0.055 |
| Niacin | -8.031 (-20.622, 4.559) | 0.208 | -0.535 ( -1.018,-0.051) | 0.031 |
| Folic acid | -35.180 (-90.542, 20.182) | 0.210 | 0.017 ( -2.465, 2.500) | 0.989 |
| Folate | -59.801 (-153.922, 34.319) | 0.210 | 0.030 ( -4.190, 4.251 | 0.989 |
| Vitamin B12 | -21.187 (-114.620, 72.245) | 0.653 | 3.346 ( -1.136, 7.828) | 0.141 |
| Vitamin D | 18.671 ( -1.296,38.639) | 0.066 | 0.527 ( -0.091, 1.146) | 0.094 |
| Vitamin K | -9.596 (-35.999, 16.807) | 0.472 | -0.353 ( -1.464, 0.759) | 0.530 |
| Vitamin C | 22.817 (-59.610,105.244) | 0.583 | -0.048 ( -2.944, 2.848) | 0.974 |

aCoeff. Estm. = adjusted coefficient estimate. Covariates in the adjusted model include age, gender, diabetes, hypertension, chronic kidney disease, congestive heart failure, and liver disease. Significance set to p < 0.006 using Bonferonni’s correction to account for multiple comparisons. Note: Although vitamin E and omega-3 usage was a part of the inclusion criteria for dietary supplements and/or CAM, the number of individuals who used these specific supplements was not enough to conduct meaningful individual analysis and thus was omitted.
