# Supplementary material for: Adults with depressive symptoms have lower odds of dietary supplement use
Source: PLoS One. 2024 May 8;19(5):e0302637. doi: 10.1371/journal.pone.0302637 (PMC11078386; doi:10.1371/journal.pone.0302637)
Supplement: S2 Table — (DOCX) [file pone.0302637.s002.docx]

**Table 2S.**

| Outcome | Depressive symptoms | | Depressive symptoms severity | |
| --- | --- | --- | --- | --- |
|  | aOR (95% CI) | *P* value | aOR (95% CI) | *P* value |
| Vitamin B family | 0.794 (0.662,0.953) | 0.014 * | 0.985 (0.977,0.993) | <0.001* |
| Fat-soluble vitamins | 0.862 (0.718,1.035) | 0.111 | 0.978 (0.970,0.986) | <0.001* |
| Water-soluble vitamins | 0.790 (0.661,0.943) | 0.010* | 0.981 (0.972,0.990) | <0.001* |

aCoeff. Estm. = adjusted coefficient estimate. Covariates in the adjusted model include age, gender, diabetes, hypertension, chronic kidney disease, congestive heart failure, and liver disease. Significance set to p < 0.017 using Bonferonni’s correction to account for multiple comparisons.
