# Supplementary material for: Adults with depressive symptoms have lower odds of dietary supplement use
Source: PLoS One. 2024 May 8;19(5):e0302637. doi: 10.1371/journal.pone.0302637 (PMC11078386; doi:10.1371/journal.pone.0302637)
Supplement: S3 Table — (DOCX) [file pone.0302637.s003.docx]

**Table 3S.**

|  | aCoef. Estm. (95% CI) | *P* value | aCoef. Estm. (95% CI) | *P* value | aCoef. Estm. (95% CI) | P value |
| --- | --- | --- | --- | --- | --- | --- |
| Vitamin B1 | 8.422 ( -4.685, 21.530) | 0.204 | -4.729 (-13.513,4.056) | 0.287 | 3.510 (-12.020,19.041) | 0.654 |
| Vitamin B2 | -2.909 ( -6.862, 1.044) | 0.147 | 0.695 (-3.531, 4.920) | 0.745 | -2.441 (-5.821,0.939) | 0.155 |
| Niacin | -2.252 ( -8.270, 3.766) | 0.458 | -6.245 (-19.946, 7.455) | 0.367 | -12.053 (-26.188,2.082) | 0.094 |
| Folic acid | -22.311 ( -78.480, 33.858) | 0.431 | -22.389 (-82.822, 38.045) | 0.463 | -42.100 (-110.121,25.920) | 0.222 |
| Folate | -37.957 (-133.426, 57.512) | 0.431 | -38.027 (-140.775, 64.720) | 0.464 | -71.577 (-187.205,44.052) | 0.222 |
| Vitamin B12 | -12.624 (-116.155, 90.906) | 0.801 | -23.930 (-119.775, 71.915) | 0.621 | -17.076 (-141.354,107.201) | 0.785 |
| Vitamin D | -1.624 (-28.977, 25.730) | 0.906 | 16.474 (-5.184,38.131) | 0.134 | 19.401 (-7.871,46.673) | 0.161 |
| Vitamin K | -3.609 (-13.403, 6.184) | 0.465 | -8.662 (-35.857, 18.533) | 0.5285 | -9.357 (-40.201,21.488) | 0.548 |
| Vitamin C | 3.175 ( -95.903,102.252) | 0.949 | 8.918 (-71.390, 89.226) | 0.826 | 40.207 (-80.546,160.959) | 0.510 |

aCoeff. Estm. = adjusted coefficient estimate. Covariates in the adjusted model include age, gender, diabetes, hypertension, chronic kidney disease, congestive heart failure, and liver disease. Significance set to p < 0.006 using Bonferonni’s correction to account for multiple comparisons. Note: Although vitamin E and omega-3 usage was a part of the inclusion criteria for dietary supplements and/or CAM, the number of individuals who used these specific supplements was not enough to conduct meaningful isolated analyses and thus was omitted.
