# Supplementary material for: Adults with depressive symptoms have lower odds of dietary supplement use
Source: PLoS One. 2024 May 8;19(5):e0302637. doi: 10.1371/journal.pone.0302637 (PMC11078386; doi:10.1371/journal.pone.0302637)
Supplement: S4 Table — (DOCX) [file pone.0302637.s004.docx]

**Table 4S.**

|  | aOR. (95% CI) | *P* value | aOR. (95% CI) | *P* value | aOR. (95% CI) | P value |
| --- | --- | --- | --- | --- | --- | --- |
| Vitamin B family | 1.172 (0.913,1.504) | 0.210 | 0.774 (0.631,0.950) | 0.015* | 0.813 (0.629,1.051) | 0.113 |
| Fat-soluble vitamins | 1.339 (1.064,1.686) | 0.014* | 0.812 (0.651,1.012) | 0.064 | 0.944 (0.744,1.198) | 0.634 |
| Water-soluble vitamins | 1.045 (0.816,1.338) | 0.725 | 0.805 (0.658,0.984) | 0.035 | 0.772 (0.609,0.979) | 0.033 |

aCoeff. Estm. = adjusted coefficient estimate. Covariates in the adjusted model include age, gender, diabetes, hypertension, chronic kidney disease, congestive heart failure, and liver disease. Significance set to p < 0.017 using Bonferonni’s correction to account for multiple comparisons.
