# Supplementary material for: Adults with depressive symptoms have lower odds of dietary supplement use
Source: PLoS One. 2024 May 8;19(5):e0302637. doi: 10.1371/journal.pone.0302637 (PMC11078386; doi:10.1371/journal.pone.0302637)
Supplement: Attachment — Submitted filename: Response letter.docx [file pone.0302637.s005.docx]

**Response to reviewers**

Reviewer #1: This manuscript utilized data from NHANES 2005-2018 to address the question whether adults with depression use nutritional supplements to a different degree than non-depressed adults. This is an interesting question as there is evidence that supplement use can be used successfully as adjunct therapy in depression. Using this large, representative sample is a strength of the study; however, researchers are limited to the data collected to direct their question.

My main concern with this work is how the outcome variables were defined (part 2.3). The investigators defined dietary supplement use by reporting individual nutrient use: B1, B2, B3, B6, B12, vitamins C, K, and D, folic acid, folate choline, calcium, P, Mg, Fe, Zn, Cu, Na, K, Se, and caffeine. Additionally, nutrients were grouped as water soluble vitamins, fat soluble vitamins, and B vitamins. CAM was defined as folate, Mg, B6, B9, D, Zn, and Se. Hence, there is tremendous overlap in the data presented, and the results, thus, are convoluted and repetitive. Also, it is unclear if multivitamin/mineral use is included in all of these categories, or if only individual supplementation is examined.

A more systematic approach (with no overlap) would produce more useful, meaningful results. Often multivitamin/mineral use is separated from single nutrient supplementation. In addition to a multivitamin/mineral supplement, the top 3-5 supplements could be investigated (with an ‘other’ group). For fat soluble vitamins, only D and K are examined – yet E is one of the leading individual nutrients supplemented in the US. The omega 3 fats are also at the top of the list of supplemented nutrients and are not examined in this report (although mentioned in the discussion).

Why is caffeine in this analysis? Furthermore, there is no data presented on caffeine as well as several other nutrients listed above. If variables are stated as outcomes in the methods section, they need to be reported in the results section.

**Response**: Thank you for your valuable feedback. We appreciate the insightful comments provided by Reviewer #1. In response to your concerns, we have made several revisions to enhance the clarity and relevance of our study.

- 1. Multivitamin/Mineral Use: We have incorporated 'multivitamin/mineral use' into our dietary supplement analysis, providing a more comprehensive perspective on supplement intake.
- 2. Inclusion of Missing Nutrients: Based on your suggestion, we have expanded our analysis to include Vitamin E and Omega-3 Fatty Acids.
- 3. Exclusion of Caffeine: We acknowledge the oversight and have removed caffeine from the analysis.
- 4. Refinement of CAM Definition: To address the issue of repetitiveness, we have redefined CAM based on the available NHANES data. We acknowledge the absence of other CAM agents including herbal agents data and, unfortunately, NHANES does not provide this information. The revised definition of CAM now includes: ‘’Selected CAM agents were: folate, magnesium, vitamin B6, B9, D, zinc, selenium, and omega-3 fatty acids. In this study, CAM was defined as a binary variable of “Yes” if any of the above CAM agents were taken or if an individual participated in >150 minutes of physical activity a week, and “No” otherwise.’’

In the text, CAM is defined (lines 79-86) as a range of medical and healthcare systems, practices, and products – including herbal agents, and exercise. Yet, in methods CAM is defined as folate, Mg, B6, B9 (which is folate – thus repetitive), D, Zn, and Se. There is no justification for this definition of CAM and it is not consistent with the accepted definition of CAM. See: https://www.cancer.gov/about-cancer/treatment/cam Also, the authors discuss the use of OTC – yet they did not look at these data in the NHANES data set. The authors need to be more cautious in their approach to the research question.

Since herbal agents, exercise, and omega 3 fats are in the NHANES data set, it is unclear why these variables were not assessed in this investigation.

**Response**: Thank you for bringing these concerns to our attention. We have redefined CAM based on the available NHANES data. We acknowledge the absence of other CAM agents including herbal agents data and, unfortunately, NHANES does not provide this information. The revised definition of CAM now includes: ‘’Selected CAM agents were: folate, magnesium, vitamin B6, B9, D, zinc, selenium, and omega-3 fatty acids. In this study, CAM was defined as a binary variable of “Yes” if any of the above CAM agents were taken or if an individual participated in >150 minutes of physical activity a week, and “No” otherwise.’’

Thank you for providing clarification on the dataset limitations related to OTC medications. We want to acknowledge your comment and clarify that the NHANES dataset specifically includes information on prescribed medications only, and unfortunately, OTC data is not available.

Other concerns:

• The introduction is sourced mainly from reviews and websites. There is only a cursory overview of the topic with little primary research cited. Depending on second-hand sources to such a large degree lowers the confidence of the reader for accepting the rational provided to justify the investigation. Include primary data in the introduction to support statements.

**Response**: Thank you for your comment. We have incorporated the original studies as references in the introduction section.

• Line 71: what is meant by ‘regular’ diet?

**Response**: Thank you for your comment. In the context of our statement, a "regular" diet refers to a balanced and nutritionally adequate diet that provides essential nutrients in appropriate proportions. We revised that section of the manuscript.

‘’Despite the fact that dietary supplements are not recommended for individuals who are healthy and consume a well-balanced and nutritionally adequate diet, the industry generates an annual revenue of 1.2 billion USD.’’

• Section 2.1: provide citations for the characteristics of NHANES data.

**Response**: Thank you for your comment. We added a citation for the characteristics of NHANES data.

‘’Zipf G, Chiappa M, Porter KS, Ostchega Y, Lewis BG, Dostal J. National Health and Nutrition Examination Survey: Plan and Operations, 1999–2010. Vital Health Stat. 2013;1(56):37.’’

• Line 105: ‘need to sign’ to replace ‘have to sign’

**Response**: We appreciate your keen observation. In response to your comment, we have made the suggested change, replacing 'have to sign' with 'need to sign'.

• Line 123: what is “249” – there is no definition or citation.

**Response**: Thank you for your comment. 249 is how NHANES coded antidepressants in the Prescription Medications - Drug Information Questionnaire questionnaire. We added ‘’<https://wwwn.cdc.gov/Nchs/Nhanes/1999-2000/RXQ_DRUG.htm>’’ to the methods section for further clarification.

• Throughout the text, do not capitalize nutrients – these are not proper nouns.

**Response**: Thank you for your comment. In response to your feedback, we have made the necessary corrections in the revised manuscript to ensure that nutrients are not capitalized

• Line 145: provide a citation

**Response**: Thank you for your comment. We provide citations for line 145 in the revised manuscript.

• All tables should have a legend to explain the presentation of the data and the p value.

**Response**: Thank you for your comment. We added legends to all tables to further explain the data.

• Table 1: For ‘sex’ add a line for male data (similar to how there is a line for each component of ‘race’).

**Respons**: Thank you for your comment. In response to your comment, we have added a dedicated line in Table 1, providing a more comprehensive presentation of both male and female participant numbers.

• Table 1: the final 6 lines need to be reconsidered as discussed above. (similar comment for tables 4-6)

**Response**: Thank you for your comment. We redefined these variables in the revised manuscript.

• Line 213: replace ‘levels’ with ‘usage’

**Response**: Thank you for your comment. In response to your comment, we have made the suggested change, replacing 'levels' with '‘usage’'.

• Discussion: the first paragraph can be deleted as it is a repeat of the results.

**Response**: Thank you for your comment. We deleted the first paragraph of discussion in the revised manuscript.

• Line 316: need to add ‘compared to adults not reporting depression’. The comparison group needs to be identified.

**Response**: Thank you for your comment. In response to your comment, we have revised the sentence on line 316 to explicitly state, ‘’In conclusion, our results indicated significantly lower odds of dietary supplement use in individuals with depressive symptoms compared to adults not reporting depressive symptoms.’’

Reviewer #2: Thank you for the opportunity to review this paper

1. The authors referenced line 81 to justify CAM's indication for depression, citing source number 14. However, upon further examination of this source, it does not distinctly indicate the role of CAM in treating depression. Referencing the article by Ng, J.Y., Nazir, Z. & Nault, H. (2020) on "Complementary and alternative medicine recommendations for depression: a systematic review and assessment of clinical practice guidelines," it was observed that the quality of CAM recommendations was generally lower. Therefore, it is crucial to narrow down the scope of CAM that has proven to be beneficial for depression.

**Response**: We appropriate the reviewer for their insightful comment. We revised that section based on your feedback: ‘’Despite the advantages associated with CAM, such as a lower incidence of adverse events, reported efficacy, a comprehensive perspective on individual problems, and dissatisfaction with traditional care, the quality of CAM recommendations for depression has been noted to be generally lower.’’

2. In reference to line 99 mentioning the use of NHANES data covering participants from 20 to 80 years old, a 2020 US report highlights varying prevalence rates of depression across age groups, with the highest among 18–24 years (21.5%) and the lowest among those ≥65 years (14.2%). However, the data analyzed in this study covers adults from 18 to 80 years old (line 166). Clarification is needed regarding the real age range within this dataset. Additionally, it's important to note that this dataset only covers information until 2018, which presents a limitation. The reason for not utilizing more current NHANES data should be addressed.

**Response**: Thank you for your insightful comment. We have addressed the concern about the age range in the revised manuscript, providing clarification on the NHANES data source. The corrected statement now reads:

‘’NHANES is a survey based on nationally representative data from civilian, non-institutionalized populations aged 18-80 years across 50 states of the United States, utilizing a complex, multistage, probability sampling design.’’

Furthermore, we acknowledge the limitation associated with the dataset's timeframe, as it covers information up to 2018. In the revised manuscript, we have explicitly mentioned the reason for not utilizing more recent NHANES data. Due to the unprecedented impact of the COVID-19 pandemic on mental health and depression, we made the decision to focus on data up to 2018 to maintain consistency and reliability in our analysis.

‘’The strength of our study is that NHANES is a survey that encompasses a representative sample of the noninstitutionalized population in the US, which adds to its credibility, it is important to note a limitation. The data utilized in our analysis only extends up to the year 2018 due to the unique circumstances presented by the COVID-19 pandemic and its impact on mental health. This time frame limitation may impact the generalizability of our findings.’’

3. Concerning the definition of MDD, the use of PHQ-9 (with a sensitivity and specificity cutoff of 10 at more than 85%) is deemed acceptable as a screening tool rather than a standardized diagnostic tool. However, it should be acknowledged that individuals screened as MDD and remaining untreated might not necessarily require antidepressants, potentially not reflecting true MDD cases. Furthermore, the tool might not distinctly capture the level of depression, which should be highlighted as a limitation.

**Response**: Thank you for your insightful comment. We appreciate the importance of clarifying the nature of the PHQ-9 as a screening tool. To accurately reflect this distinction, we have modified instances of 'depression' to 'depressive symptoms' throughout the manuscript, including the title. Additionally, we have incorporated this clarification into the limitation section of the manuscript to transparently communicate the nature of our assessment tool and its implications for the study's findings:

‘’In addition, regarding the definition of depressive symptoms, our study employed the PHQ-9 as a screening tool, utilizing a sensitivity and specificity cutoff of 10 at more than 85%. While deemed acceptable for screening, it's crucial to acknowledge that the PHQ-9 serves as a screening tool rather than a standardized diagnostic tool. Consequently, individuals identified as MDD through screening, who remain untreated, may not necessarily require antidepressants, potentially not reflecting true MDD cases. Furthermore, the tool might not distinctly capture the level of depressive symptoms, representing a limitation in our study.’’

4. Further examination of the CAM definition reveals that it encompasses more than just dietary supplements, contradicting the initial understanding. Notably, at line 133, B6, vitamin D, and Zinc are also considered part of CAM. To maintain consistency throughout the manuscript, a single term, preferably "dietary supplement," should be used instead of alternating between CAM and dietary supplement. Please refer to reference at question 1 for the various definition of CAM.

**Response**: Thank you for your comment. We have redefined CAM based on the available NHANES data. We acknowledge the absence of other CAM agents including herbal agents data and, unfortunately, NHANES does not provide this information. The revised definition of CAM now includes: ‘’Selected CAM agents were: folate, magnesium, vitamin B6, B9, D, zinc, selenium, and omega-3 fatty acids. In this study, CAM was defined as a binary variable of “Yes” if any of the above CAM agents were taken or if an individual participated in >150 minutes of physical activity a week, and “No” otherwise.’’

5. Certain variables within the NHANES dataset might act as confounders. For instance, the presence of chronic diseases like diabetes could influence the decision to take dietary supplements rather than using them specifically for treating depression. It's essential to explore whether the authors investigated other potential covariates or confounders that are available in the dataset.

**Response**: Thank you for your comment. In response, we have expanded our analysis by incorporating additional covariates (chronic diseases). The updated set of covariates now includes age, gender, diabetes, hypertension, chronic kidney disease, congestive heart failure, and liver disease.

6. The method for defining depression severity is unclear. Specifically, how did the authors delineate the severity using the raw total score of PHQ-9? What cutoffs were employed to categorize mild, moderate, and severe depression? Line 155 mentions analyzing depression severity using logistic regression. Clarification is needed on the cutoffs and grouping used for the analysis of dietary supplement and depression severity.

**Response**: Thank you for your comment. We did not categorize depressive symptoms severity into mild/moderate/severe due to small sample sizes. We added ‘’Depressive symptoms severity was defined as just the raw PHQ-9 score, where a higher score indicates more depressive symptoms.’’ to further clarity how we defined depressive symptoms severity.

7. Due to the unclear definition of certain variables, it would be inappropriate to draw conclusions solely based on the results.

**Response**: Thank you for your insightful comment. In response, we have taken steps to enhance the clarity of our study by redefining certain variables, specifically within the realm of CAM. Additionally, we have incorporated additional covariates to strengthen the robustness of our results. These refinements aim to address concerns about variable definition ambiguity, ensuring a more reliable foundation for our conclusions.
