# Supplementary material for: Adults with depressive symptoms have lower odds of dietary supplement use
Source: PLoS One. 2024 May 8;19(5):e0302637. doi: 10.1371/journal.pone.0302637 (PMC11078386; doi:10.1371/journal.pone.0302637)
Supplement: Attachment — Submitted filename: Response to reviewers.docx [file pone.0302637.s006.docx]

**Response to reviewers**

1. It is still essential to use the recent data. If the authors are concern about the COVID, it can be adjusted with statistical method treating COVID as confounding or making a subgroup analysis of this nested data for more relevant and novel result.

**Response**: Thank you for your valuable feedback. We appreciate your suggestion regarding adjusting for COVID effects using statistical methods. However, the data is not avaible (please see: 2019-2020:not representative- [https://wwwn.cdc.gov/nchs/nhanes/continuousnhanes/default.aspx?BeginYear=201](https://wwwn.cdc.gov/nchs/nhanes/continuousnhanes/default.aspx?BeginYear=2019); 2017-March 2020 Pre-pandemic: Not completed- <https://wwwn.cdc.gov/nchs/nhanes/continuousnhanes/default.aspx?Cycle=2017-2020>; 2021-2023:Not available- <https://wwwn.cdc.gov/nchs/nhanes/continuousnhanes/default.aspx?Cycle=2017-2020>). Furthermore, we believe that adjusting for all potential COVID effects might not be feasible due to several reasons. Firstly, the NHANES dataset does not include specific questions regarding the history of COVID-19 infection or treatments received. Secondly, COVID-19 has had multifaceted impacts on health outcomes, including indirect effects on lifestyle, mental health, and healthcare access, which are not easily quantifiable within the NHANES dataset. Additionally, using data after the COVID-19 period might introduce further challenges. Post-COVID data may be confounded by temporal changes in lifestyle and health-seeking behavior, limited availability of such data depending on the timing of the study, continued uncertainty and variability of pandemic effects, potential shifts in dietary patterns during the pandemic, the profound impact of the pandemic on mental health, and ethical considerations surrounding research conducted immediately after a major global health crisis.

2. Table 1 one elaborates on the characteristic of the participants. It is obvious that those people with chronic disease tend to have depressive symptom, However it was not briefly discussed in the result and discussion section. Just add some statement that using CAM and dietary supplement may also have interaction with the chronic disease medication.

**Response**: Thank you for your comment. We added ‘’Additionally, It is noteworthy to consider that participants with chronic diseases in this study exhibited higher levels of depressive symptoms. The use of CAM and dietary supplements among these individuals may introduce interactions with their chronic disease medications, which could contribute to the observed depressive symptomatology. Future research should delve deeper into these potential interactions to better understand their impact on mental health outcomes.’’ to the discussion section.

3. It It will be more meaningful to classify the severity of disease rather than using the raw score of the PHQ9 so the aOR would be more meaningful to translate. As a reader will prefer to see the aOR of severity level rather than aOR for each 1 unit of PHQ9. Table 2,4 and 5 on depressive severity have aOR of around 0.9 which is quite smaller. To accommodate this, logistic regression is preferred. We might see more meaningful association of particular CAM and supplement such as vitamin D

**Response**: Thank you for your comment. We first categorized depressive symptoms into 'Yes (at risk)' and 'No,' in hopes of simplifying the understanding of the risk of depressive symptoms and CAM intake. We agree that additional insight can be drawn with severity of disease though it was not possible to run such models reliably due to the small number of individuals in some of our categories. Instead we had included the PHQ9 as a continuous score to allow us to observe trends without losing statistical power that may have happened when dichotomizing our depressive symptom scores.

4. Also the authors talked about depressive symptoms which is treated as binary using cutoff of 10 (<10 is no and >10 is yes). Why didn't the authors used logistic regression for table 4 and 6 and just present the odd ratio of table 5 and 7 to make the manuscript more concise without abundant coefficients tables?

**Response**: Thank you for your comment. We would like to clarify that we did not use logistic regression for Table 4 and 6 as the outcome was specific vitamin intake (ex. mg, mcg) which were treated as continuous variables. Linear regressions were used for each vitamin intake where depressive symptoms were included as one of the covariates. Based on your comment, we moved tables 4-7 to supplementary materials to avoid having abundant tables.

5. In characteristic table 1, age was not different between group, but race was different. Why the adjusted model did not use race but put age instead?

**Response**: Thank you for your comment. We added ethnicity to covariates and re-analyzed the data.

6. Is it still fall into study objective if the authors compare the treated individual vs non depressive individual, which is missing in the manuscript?

**Response**: Thank you for your comment. We compare the treated individual vs non depressive individual analysis results to the revised manuscript.

‘’No statistically significant association was seen between those with treated depressive symptoms versus no depressive symptoms in terms of dietary supplement usage (Table 3).’’

‘’In those with treated depressive symptoms compared to no depressive symptoms, CAM use was lower in comparison (aOR = 0.763, 95% CI = 0.598,0.973, p = 0.030) (Table 3).’’

We also added ‘’Our study's findings suggested a notable difference in CAM usage between individuals with treated depressive symptoms and those without. This indicated that individuals with treated depressive symptoms may be less inclined to turn to CAM therapies compared to individuals without depressive symptoms. One possibility is that individuals receiving antidepressant treatment for depressive symptoms, may feel that these conventional methods are sufficient for managing their symptoms. They might prioritize the guidance of healthcare professionals and evidence-based antidepressants over CAM approaches. Additionally, the stigma often associated with mental health conditions such as depressive symptoms could play a role. Some individuals might be more comfortable discussing and seeking treatment for their depressive symptoms within the context of traditional healthcare settings, rather than exploring CAM options. Further research should explore the reason for this phenomenon.’’ to the discussion section of the revised manuscript.

7. Again, this manuscript reveals that adult with depressive symptoms have lower use of cam and supplement, although in the introduction, some CAMs are not clinically recommended. Looking at table 6 and 7 the intake of fat soluble vitamin which also cover vitamin D was significantly higher in treated depressive individual Which is good. With these conflicting results, what would be the final conclusion and recommendations?

**Response**: Thank you for your comment. Based on your comment we added ‘’In conclusion, our results indicated significantly lower odds of dietary supplement use in individuals with depressive symptoms compared to adults not reporting depressive symptoms. Notably, individuals receiving treatment for depression demonstrated increased usage of dietary supplements, particularly fat-soluble vitamins such as vitamin D compared with those with depressive symptoms who were not taking antidepressants. Conversely, those with untreated depressive symptoms had lower odds of CAM use compared with participants without depressive symptoms. These findings suggest that treatment may influence supplement intake, especially for essential vitamins. Further replication and validation is needed along with definitive studies to further evaluate the underlying reasons for this phenomenon among individuals with depression. Healthcare providers should engage in discussions regarding the potential benefits of dietary supplements and CAM with patients experiencing depressive symptoms, particularly those not undergoing treatment. Clinicians should consider the impact of treatment on vitamin intake, specifically fat-soluble vitamins, and potentially monitor or recommend appropriate supplementation for individuals with depressive symptoms to ensure adequate nutritional support.’’ to the revised manuscript.
